# SCOUT Radar Localization System‐Guided Excision of Nonpalpable Breast Lesions: Initial Experience in a Latin American Population

**DOI:** 10.1155/tbj/6764918

**Published:** 2026-11-10

**Authors:** Moises Cukier, Sara Pons, José Espino, Maylin Ruiz

**Affiliations:** ^1^ Department of Surgery Hospital Pacífica Salud Panama City Panama; ^2^ Faculty of Medicine University of Panama Panama City Panama; ^3^ Hospital Pacifica Salud Panama City Panama

**Keywords:** nonpalpable breast lesion, SCOUT Radar Localization

## Abstract

**Background and Aims:**

SCOUT Radar Localization is a wireless technology that is an increasingly preferred method to localize nonpalpable breast lesions. As the use of this wireless system has yet to be described in Panama, this study was conducted to assess the feasibility of its use in the localization and excision of nonpalpable breast lesions.

**Methods:**

This prospective, observational study was conducted at a single center in Panama. Patients > 18 years with nonpalpable breast lesions who underwent wireless localization using SCOUT Radar Localization between April 2025 and February 2026 were analyzed. Key study measures included time between SCOUT placement and surgery, surgical margins, lesion size, based on imaging and pathology assessment, and presence of complications.

**Results:**

Twenty patients (mean age: 58.7 ± 12.9 years) with nonpalpable breast lesions (including malignant, premalignant, and high‐risk) were analyzed. SCOUT reflectors were successfully placed in all patients. The mean time between reflector placement and surgery was 2.5 (±0.5) days for most patients (*n* = 18). Among the two patients who deferred surgery (by 49 days and 213 days), device localization and removal were not impacted. All surgical interventions were performed by a single breast surgical oncologist. Surgical margins were negative for all cases and there was consistency between lesion size determined by imaging (1.09 ± 0.34) vs. pathology assessment (1.41 ± 0.36) cm. No complications were observed.

**Conclusions:**

Findings from this study suggest that SCOUT is a safe, effective, and convenient method to manage nonpalpable breast lesions (localization and resection). The high rate of technical success may help broaden its use in Latin America.

**Highlights**
- First reported experience of SCOUT Radar Localization in Latin America.
- Use of SCOUT was found to be a safe and effective method involved in the localization and resection of nonpalpable breast lesions.
- The clinical benefits associated with SCOUT’s use offer advantages over traditional wire‐guided localization methods.

## 1. Introduction

Breast cancer rates have consistently increased throughout Latin America over the last 2 decades [1]. Early detection of breast lesions, particularly those that are nonpalpable can significantly improve a patient’s prognosis [2].

Historically, wire‐guided localization has been the mainstay approach for nonpalpable breast lesion localization [3]. Despite its low‐cost appeal, mounting disadvantages, such as patient discomfort, high complication rates, and logistical challenges associated with the need to schedule surgery soon after wire placement created an impetus for newer, wireless technologies [4–8].

One example of an increasingly preferred wireless technology is the SCOUT Radar Localization System. As a nonradioactive, nonmagnetic, preoperative lesion localization technology, SCOUT is associated with several well documented advantages [9]. The device used to localize lesions is a standard nitinol reflector (12 mm in length) that consists of two antennae to help anchor the device within the lesion, an infrared light receptor, and a transistor switch. The reflector is placed under mammography or ultrasound guidance [8]. The location of the reflector (and thus lesion) is detected using an accompanying radar‐emitting probe and console that processes the signal. The inert nature of the reflector is comfortable for patients and allows for the device to be placed any time prior to surgery, which is highly convenient for both patients and physicians.

To date, several studies have documented the practical advantages of SCOUT, including its favorable safety profile and high rate of technical success [10–15]. However, use of this technology is not common in Panama and throughout Latin America. Based on the increased prevalence of breast cancer in this region and the known prognostic benefits associated with early detection of nonpalpable lesions, this single‐center study was conducted to confirm the feasibility and clinical utility of SCOUT’s use to help promote wider adoption of a technology that has the potential to meaningfully improve patient care and outcomes.

## 2. Materials and Methods

### 2.1. Study Design

This was an observational, prospective study conducted at Pacífica Salud Hospital in Panama City, Panama. Ethics board approval from the CBI of Pacifica Salud (Approval number 149, March 26, 2026) was obtained prior to commencement of the study. All patients provided written and informed consent. This study complied with the Declaration of Helsinki.

Artificial Intelligence was not used in the design of this study.

### 2.2. Patients

Patients were eligible for inclusion in the study if they were > 18 years, had nonpalpable breast lesions (including malignant, premalignant, and high‐risk), and were scheduled to undergo wireless localization using SCOUT Radar Localization (Merit Medical Systems, Inc., South Jordan, UT, USA) between April 2025 and February 2026. Patients with incomplete medical record data were excluded.

### 2.3. Localization Procedure

A single interventional breast radiologist performed the preoperative placement of the SCOUT reflector guided by ultrasound or mammogram. In all cases, a standard 12 mm reflector was inserted using a 16 G introducer needle. In one patient, who was a candidate for neoadjuvant therapy (Figure 1a), two reflectors were placed to help demarcate the lesion prior to initiation of the regimen (Figure 1b).

**FIGURE 1:**
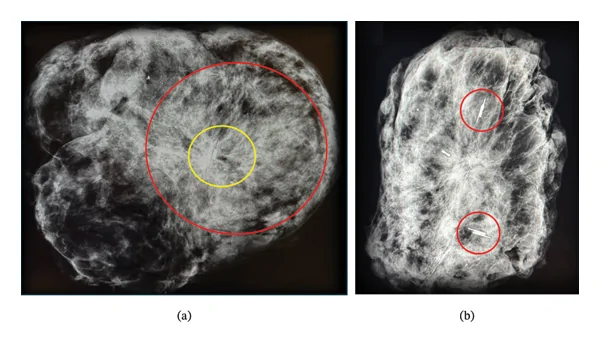
Mammography of SCOUT reflectors. (a) Mammography of a patient with locally advanced breast cancer who was a candidate for neoadjuvant therapy. Preneoadjuvant tumor size (red circle) and residual size postneoadjuvant therapy (yellow circle). The SCOUT reflector was placed prior to treatment, enabling localization of the residual lesion during surgery and (b) mammogram demonstrating the bracketing technique with two SAVI SCOUT reflectors (red circles).

Intraoperatively, the SCOUT probe was used to confirm the reflector position within the breast, to define the incision site, and to confirm the distance to margins in the resected specimen. All interventions were performed by a single breast surgical oncologist with over 15 years of experience.

### 2.4. Study Measures

Study measures of interest included patient demographics, lesion characteristics, imaging modality at the time of device placement, time to surgery following device placement, radiological verification, modality of verification, and administration of neoadjuvant therapy. Surgical outcomes described included surgical margin status (positive or negative), re‐excision, lesion size based on imaging, and pathological assessment. Device migration and the incidence of complications were also reported.

### 2.5. Analysis

Study measures were summarized using descriptive statistics, with mean ± standard deviation (SD) used for continuous variables and proportions used for categorical variables. As this analysis was purely descriptive, no hypothesis testing was conducted.

## 3. Results

Among the 20 patients with nonpalpable lesions included in this study, on final pathology report, seventeen (85%) had malignant lesions (88% invasive ductal and 12% invasive lobular), two (10%) lobular in situ carcinoma, and one (5%) ductal in situ carcinoma (Table 1).

**TABLE 1:** Clinical characteristics.

Characteristic	Patients (*N* = 20)
Age, mean (SD)	58.7 (12.9)
Sex, *n* (%)	
Female	20 (100)
Histological subtype, *n* (%)	
Premalignant lesions	3/20 (15)
In Situ Ductal	1/3 (3.3)
In Situ Lobular	2/3 (6.7)
Malignant lesions	17/20 (85)
Invasive Ductal	15/17 (88.2)
Invasive Lobular	2/17 (11.8)
Invasive + In Situ Ductal	1/17 (5.9)
Palpable lesion, *n* (%)	
Yes	0 (0)
No	20 (100)

Abbreviation: SD, standard deviation.

Ultrasound was the most common imaging modality used to place SCOUT reflectors (*n* = 19, 95%). Mammogram was the placement modality used in only one patient. Eighteen patients had reflectors placed and proceeded to surgery as scheduled. Among these 18 patients, the mean (±SD) number of days between reflector placement and surgical excision was 2.5 (±0.5) days. Two patients experienced delays between device placement and surgery. One patient experienced a delay of 49 days between reflector placement and surgery due to an unexpected cardiac event following anesthesia that was unrelated to the device. For this patient, the option to defer the surgical date until resolution of the cardiac issue was provided. Following resolution of the cardiac issue, the patient underwent surgery without incident. The second patient who experienced a delay between reflector placement and surgery was due to neoadjuvant therapy. For this patient the time between reflector placement and surgery was 213 days (Table 2).

**TABLE 2:** SCOUT placement method.

**Variables**	**Patients (*N* = 20)**	

Image‐placement method, *n* (%)		
Ultrasound	19 (95)	
Mammography	1 (5)	

**Placement-surgery time**		**Days, mean (±SD)**

Standard cases (*n*)	18	2.5 ± 0.5
Delayed surgery case (*n*)	1	49
Neoadjuvant therapy case (*n*)	1	213
Radiological verification after resection, *n* (%)		
No	19 (95)	
Yes (mammogram)	1 (5)	
Intraoperative verification before and after resection, *n* (%)		
SCOUT probe	20 (100)	

Abbreviation: SD, standard deviation.

Intraoperative verification was performed in all cases and documented in the operative report. Before the incision, the probe confirmed the location and position of the reflector in the breast. Following resection, the probe was used to analyze, ex‐vivo, the specimen and confirm reflector removal, as well as margin distance around the edges of the lumpectomy.

Surgical margins were negative for all patients (i.e., no tumor at ink for invasive carcinomas and > 2 mm for in situ carcinomas). The mean (±SD) lesion size on imaging versus pathology examination for the 19 patients who did not undergo neoadjuvant therapy was 1.09 (±0.34) vs. 1.41 (±0.36), a difference of 0.29 (±0.35). For the patient who underwent neoadjuvant therapy, the mean (±SD) lesion size on imaging versus pathology examination was 3.3 vs. 1.3 cm, a difference of 2.0 cm (Table 3).

**TABLE 3:** Surgical outcomes.

Outcome variable	Patients (*N* = 20)
Surgical margins, *n* (%)	
Negative	20 (100)
Positive	0 (0)
Lesion size‐imaging (cm), mean ± SD	
No neoadjuvant therapy	1.09 (±0.34)
Neoadjuvant therapy	3.3
Lesion size‐pathological (cm), mean ± SD	
No neoadjuvant therapy	1.41 (±0.36)
Neoadjuvant therapy	1.3 cm
Lesion size‐imaging vs pathological (cm), mean ± SD	
No neoadjuvant therapy	0.29 (±0.35)
Neoadjuvant therapy	2.0 cm

Abbreviation: SD, standard deviation.

For the patient who underwent neoadjuvant therapy, changes in tumor size were monitored prior to treatment and after (Figure 1a). A bracketing technique was successfully used to more precisely capture lesion perimeter (Figure 1b).

No patient experience complications related to reflector placement or removal, and no device migration was observed in the two patients whose device removal was delayed.

## 4. Discussion

This study described the safety and effectiveness of SCOUT Radar Localization and resection of nonpalpable malignant breast lesions at a single center in Panama. While prior evidence exists to support the use of SCOUT in this patient population, this work is the first to describe the technology’s use in Latin America. The absence of device‐related complications, successful placement in all patients, coupled with negative margins underscores the clinical utility of this technology. The fact that these favorable results were achieved at a single center may help to encourage broader use of this technology in Latin America.

The positive results from this analysis align with prior evidence highlighting the safe and effective use of SCOUT for nonpalpable lesion localization [10–15]. For example, a systematic review and pooled analysis of over 800 cases found that SCOUT was associated with a 99.64% technical success rate (i.e., successful placement and retrieval) [10]. Moreover, re‐excision rates associated with SCOUT are generally low, 5%–10% [11, 12]. In the present study, margin positivity was 0%, which may be reflective of improved training as it relates to operator placement, with a coordinated collaboration between the interventional radiologist and breast surgeon.

One of the most commonly cited advantages of SCOUT is streamlined patient care as evidenced by the ability to decouple reflector placement from surgery. Specifically, the relief from the required same‐day scheduling, common with wire‐guided localization, has been reported to improve workflow by alleviating scheduling challenges, and avoid patient discomfort. While SCOUT can be placed any time in advance of surgery, this analysis offers specific insight regarding preoperative placement intervals. In this study, most patients proceeded to surgery, on average 2.5 days, following device placement. However, for the two patients who required a delay in their surgery (either due to completing a neoadjuvant regimen, or an unforeseen complication unrelated to the device), the device was able to remain in place longer than 30 days (the former length of time referenced [8]) with no adverse impact on the patient or device retrieval. Evidence regarding long‐term placement of SCOUT remains an ongoing need.

This analysis provides an important framework to support the adoption of SCOUT in Latin America. In addition to validating the feasibility of the successful use of this technology in a single center, this analysis provides necessary insight regarding its effectiveness in complex circumstances. For example, as previously mentioned, two of the 20 patients required delays in surgery, a 49‐day delay in one patient, and a 7.1‐month delay in another. Additionally, in one patient, more than one reflector was placed (bracketing), which is considered a complex technique given the potential for challenges associated with accurate localization [16]. The two SCOUT reflectors were separated by 3 cm and independent signal was measured perpendicular to the reflector. No issues were encountered during resection of bracketed devices in this one reported case.

The documented success of SCOUT’s use in this study provides a strong foundation for additional analyses regarding use of this technology. For example, while the current study focused on patients with nonpalpable malignant and premalignant lesions, an evaluation of SCOUT’s use in patients with nonmalignant lesions would provide a comprehensive understanding of this technology, rather than limiting its scope to oncological lesions. In this study, the standard 12 mm reflector was used on all patients. However, as this technology continues to evolve with the emergence of the 8 mm reflector, it will be important to evaluate the next generation of the technology given the anticipated benefit for patients with nonpalpable lesions. Finally, while this analysis provides key evidence supporting the streamlined device retrieval, irrespective of extended delays between placement and surgery, this should be formally explored in a real‐world population as this will help address an ongoing need.

There are some limitations of this study that must be acknowledged. First, this analysis was performed on a small sample of privately insured patients, as a result, widespread inferences cannot be made. While SCOUT can be used to localize nonmalignant lesions, this analysis only included patients with malignant and premalignant breast lesions as reimbursement for the procedure was restricted by insurance coverage criteria. Another limitation of this analysis was that it included a single cohort of patients, and as a result, no comparative analyses regarding the magnitude of clinical benefits could be assessed. Relatedly, no hypothesis testing was conducted, therefore, any magnitude of difference in margins, improved workflow, and patient‐reported outcomes could not be evaluated. While a strength of this work was that all procedures were performed by the same breast surgical oncologist, which eliminated potential variability in outcomes, because this is a single center and single operator study, the impact of the learning curve associated with the procedure could not be evaluated. However, it is noteworthy that all cases described herein were the operator’s first experience with SCOUT placement, thereby reflecting the ease of the system’s use. Despite the limitations noted in this study, this work provides valuable insight regarding the feasibility of SCOUT’s use in Panama, which can be helpful for other centers in the region looking for technologies to more effectively streamline patient care and improve patient outcomes.

## 5. Conclusion

This work demonstrated that use of SCOUT Radar Localization is a safe and effective method for the localization and excision of nonpalpable breast lesions. The functionality of the reflectors was not impacted by procedural delays or whether patients underwent neoadjuvant therapy. This flexibility, coupled with the high rate of technical success, offers convenience for patients and providers. As the first report of SCOUT’s use in real‐world practice in Latin America, this work may help to support broader adoption in this region, further improving the ability to streamline patient care and improve patient outcomes.

## Funding

The authors have nothing to report.

## Disclosure

A synopsis of this work was presented at Cancer Panama 2026, held in Colon City, Panama. Editorial assistance was provided by Merit Medical.

## Conflicts of Interest

The authors declare no conflicts of interest.

## Data Availability

The authors confirm that the article contains all relevant information.
